# Cardiopulmonary Exercise Testing in Patients with Cardiac Pacemakers: From Device Optimization to Individualized Cardiac Rehabilitation

**DOI:** 10.3390/jcm15197394

**Published:** 2026-09-23

**Authors:** Bogdan Caloian, Carmen Silvia Caloian, Raluca Tomoaia, Diana Andrada Irimie, Florina Iulia Fringu, Dan Horatiu Comsa, Gabriel Laurentiu Cismaru, Gabriel Nicolae Gusetu, Radu Ovidiu Rosu, Dana Pop

**Affiliations:** 1 Department of Cardiology-Rehabilitation, Faculty of Medicine, University of Medicine and Pharmacy “Iuliu Hatieganu”, 400347 Cluj-Napoca, Romania; raluca.tomoaia@gmail.com (R.T.); gurzaudiana@yahoo.com (D.A.I.); florina.fringu@yahoo.com (F.I.F.); dh.comsa@gmail.com (D.H.C.); gabi_cismaru@yahoo.com (G.L.C.); gusetu@gmail.com (G.N.G.); rosuradu1053@gmail.com (R.O.R.); pop67dana@gmail.com (D.P.); 2 Department of Periodontology, Faculty of Dental Medicine, University of Medicine and Pharmacy “Iuliu Hatieganu”, 400347 Cluj-Napoca, Romania; caloiancarmen@yahoo.com

**Keywords:** cardiac rehabilitation, cardiopulmonary exercise testing, cardiac pacemaker, chronotropic incompetence, rate-responsive pacing, exercise prescription, oxygen consumption, exercise capacity

## Abstract

Permanent cardiac pacing abolishes symptomatic bradycardia, yet a substantial proportion of recipients remain limited on exertion. Chronotropic incompetence is present in about half of pacemaker-wearing patients when assessed by the Wilkoff index, and the rate-responsive function intended to correct it is frequently left at the settings applied at implantation. Read across the whole cardiopulmonary exercise test rather than at peak effort, the relationship between heart rate and oxygen consumption distinguishes an inadequate sensor slope, an activity threshold set too high, a reaction time out of step with the onset of exercise, and an upper rate limit reached prematurely, each with a distinct correction. Reprogramming alters the result of the test within 24 h, so the settings should be corrected before rehabilitation begins. The objective is not the highest achievable rate but the individual ceiling above which the ventricle no longer converts frequency into output. Because heart rate is partly device-determined, training intensity should be anchored to the ventilatory threshold, to the corresponding workload and to perceived exertion, with heart rate being monitored for safety rather than used to set intensity. The aim of this review is to describe how cardiopulmonary exercise testing guides device reprogramming and training prescription in the pacemaker-wearing patient.

## 1. Introduction

A cardiac pacemaker not only prevents the heart rate from dropping below a programmed limit but can also have an important influence on the exercise capacity of the patient, as the pacing mode determines the atrioventricular synchronicity (single-chamber vs. dual-chamber devices) and the way in which the heart rate adapts to physical effort (rate-responsive function, upper rate limit, and mode-switch function) [1,2,3].

Most patients who receive a pacemaker for sick sinus syndrome or for atrioventricular block need to be enrolled in a cardiac rehabilitation programme in order to improve their exercise capacity and their quality of life. Physical training is one of the most important components of such a programme, and it has to be individualized for each patient by taking into account age, gender, comorbidities, chronic medication and fitness level [4].

The first step in prescribing a physical training plan is the evaluation of maximum exercise capacity through a standardized exercise test. In the pacemaker-wearing patient, however, a simple exercise stress test does not answer all the questions raised at the bedside: the heart rate might not adapt to effort in patients with chronotropic incompetence; the upper rate limit can be reached before the patient is limited by symptoms, with subsequent loss of atrioventricular synchronicity [5]; ischaemic or arrhythmic changes are difficult to recognize on an electrocardiogram recorded during paced atrial or ventricular beats. Cardiopulmonary exercise testing (CPET) offers more than the classical exercise test in these situations because it measures the response of the cardiovascular, respiratory and muscular systems together and because the relationship between heart rate and oxygen consumption recorded throughout the test describes the behaviour of the device under effort [6].

Chronotropic incompetence [7] is frequently encountered in this population, being present in about half of pacemaker recipients in the largest cohort investigated by cardiopulmonary exercise testing [8]; its definition, prevalence across pacing indications and functional consequences are examined in Section 3.1.

The rate-responsive function was designed to compensate for this deficit, but the results reported in the literature are discordant, with a meta-analysis of randomized trials showing a clear gain in patients without heart failure but little or none in those with heart failure [9]; the magnitude of these effects and their implications for programming are considered in Section 6.4. Activating a sensor is therefore not, by itself, a therapy; whether the resulting heart-rate profile matches the metabolic demand of a particular patient is a different question and one that the nominal settings chosen in the implantation laboratory cannot answer.

The programming decisions situated above the sensor are equally important. The upper rate limit determines the moment at which atrioventricular synchronicity is lost during effort, and the way in which this limit should be established was studied directly by Kindermann et al. [5]. The ventricular activation sequence must also be taken into account, as the 2021 European Society of Cardiology guidelines on cardiac pacing address the prevention of pacing-induced cardiomyopathy and describe the place of His bundle and left bundle branch area pacing [1], with the indications of conduction system pacing being subsequently revised by Glikson et al. in a dedicated consensus document [10]. However, the he behaviour of the pacing site during effort remains much less studied than its effect on the resting ejection fraction.

Exercise capacity is not a secondary endpoint in these patients. An impaired chronotropic response to exercise testing predicts mortality independently of the classical risk markers [11], and exercise-based rehabilitation is recommended in the European prevention guidelines across the spectrum of cardiovascular disease [12]. In spite of this, rehabilitation programmes usually treat the implanted device as if it were physiologically neutral, and training intensity continues to be prescribed as a percentage of maximum or reserve heart rate. This method assumes an intact and predictable chronotropic response, which is exactly the assumption that fails when the heart rate is dictated, either entirely or in part, by an algorithm.

Small single-centre studies, including our own, have shown that the pacing mode influences exercise capacity and that the effect of reprogramming is measurable within a short interval [13,14]. Most of the studies published in this field have enrolled a limited number of patients and returned discordant results, and the literature connecting device programming, cardiopulmonary exercise testing and the rehabilitation programme remains fragmented: electrophysiological studies rarely follow the patient into the rehabilitation programme, while rehabilitation studies rarely report and even more rarely modify the settings of the device.

The purpose of the present review was to bring these elements together: to describe the reasons for which exercise capacity remains limited in the pacemaker-wearing patient, the manner in which the cardiopulmonary exercise test should be performed and interpreted when the heart rate is determined by the device, the way in which the results of the test can be translated into individualized reprogramming, and the use of the same measurements in prescribing physical training. We also propose a sequential algorithm linking the baseline cardiopulmonary exercise test, the targeted reprogramming, the control test and the establishment of training zones, and we indicate the situations in which the available evidence is insufficient to formulate firm recommendations. In relation to existing guidance, the multicomponent rehabilitation of device recipients, the use of cardiopulmonary exercise testing, the knowledge of the programmed rate limits and ventilatory-threshold-based training are already supported by the 2021 EAPC/EHRA consensus document [15]; the distinctive contribution intended here is the systematic use of the morphology of the heart rate-to-oxygen consumption relationship to identify programming abnormalities that are individually correctable, together with the explicit separation, throughout the text, of recommendations supported by guidelines or randomized trials from strategies proposed by the authors.

The present review is limited to the interaction between the pacing system, the response to effort and the rehabilitation programme. The diagnostic use of the cardiopulmonary exercise test in the differentiation between physiological and pathological cardiac adaptation was discussed elsewhere [16].

## 2. Methods

### 2.1. Literature Search and Study Selection

We searched PubMed (National Library of Medicine, Bethesda, MD, USA), Scopus (Elsevier, Amsterdam, The Netherlands) and Web of Science (Clarivate, Philadelphia, PA, USA) for English-language articles published up to January 2026, combining terms related to cardiopulmonary exercise testing (“cardiopulmonary exercise testing”, “CPET”, “oxygen consumption”, “VO_2_”, “oxygen pulse”, “VE/VCO_2_”, “ventilatory threshold”, and “anaerobic threshold”) with terms related to cardiac pacing and rehabilitation (“cardiac pacemaker”, “rate responsive pacing”, “rate-adaptive pacing”, “chronotropic incompetence”, “upper rate limit”, “cardiac resynchronization therapy”, “implantable cardioverter-defibrillator”, “cardiac rehabilitation”, “exercise training”, and “exercise prescription”). The reference lists of the key articles and of the current European and American guidelines were screened for additional sources. The last search was run in January 2026. Only publications in English were considered; records identified in other languages were noted but not formally enumerated, and this language restriction is acknowledged among the limitations in Section 9.3. As a narrative rather than a systematic review, the work did not follow a registered protocol and does not include a PRISMA flow diagram or a formal count of screened records.

A narrative review has no protocol to fall back on, so it is worth setting out how we decided what to include. Titles and abstracts were screened by two authors (Bogdan Caloian and Diana Andrada Irimie), and the full text of anything that appeared relevant was read by at least two of the authors. Disagreements were resolved by discussion within the author group rather than by any formal adjudication rule. We considered original studies, meta-analyses, consensus documents and society guidelines. Case reports were used sparingly—and only where they illustrated a mechanism or an entire clinical sequence that larger studies had not addressed. Studies were considered eligible if they reported cardiopulmonary or exercise data in adults carrying a permanent pacemaker or an implantable cardioverter–defibrillator or a cardiac resynchronization device or if they addressed the programming, rehabilitation or exercise prescription of such patients; mechanistic studies in other populations were retained only where they established a principle later applied to device recipients. Reports were not used to support quantitative statements if they did not describe the exercise-testing method, if the pacing mode and sensor status during testing could not be determined, or if the full text was unavailable in English.

Relevance alone did not determine which papers carried weight in the discussion. Where several studies addressed the same question, we gave preference, broadly in this order, to work that recruited pacemaker or device recipients rather than general heart-failure or cardiology populations; to larger or multicentre cohorts over single-centre series; to prospective and randomized designs over retrospective ones; and to more recent studies where pacing technology, sensor design or programming practice had changed materially since the earlier work. Whether the exercise data were obtained by gas exchange rather than by a simple exercise test and whether the device settings under which the patient was tested were reported mattered to us as much as sample size. Reports that gave cardiopulmonary values without describing the exercise protocol, without stating whether maximal effort had been achieved, or without recording the pacing mode and sensor status during the test were read but were not used to support any quantitative statement.

We did not apply a formal quality-appraisal instrument, and we make no claim that the resulting selection is exhaustive. When studies disagreed, our practice was to present both findings and to identify the population each came from rather than to reconcile them or to adopt the more convincing result. The question of whether augmenting the paced heart rate improves exercise capacity, where the evidence in patients without heart failure and the evidence in heart failure point in opposite directions, is handled in that way in Section 3.4 and Section 6.4. Where a single study or a single case underpins a statement, we tried to make that visible in the text. The limitations of this approach are considered further in Section 9. Throughout the review, the strength of the evidence behind each statement is indicated in the text: randomized trials and meta-analyses are distinguished from small single-centre series, case reports and physiological reasoning, and recommendations supported by current guidelines are distinguished from strategies proposed by the authors, the latter being identified as such.

### 2.2. Use of Artificial Intelligence in Manuscript Preparation

During the preparation of this manuscript, the authors used Claude (Anthropic, San Francisco, CA, USA; Claude Sonnet 5 and Claude Opus 4.8 models), a large language model-based AI assistant, at several stages of the writing process: (i) to retrieve and screen candidate references identified through the literature search described in Section 2.1; (ii) to draft and structure the narrative text of each section based on the direction, clinical framing and interpretive judgment provided by the authors; (iii) to generate the two schematic figures using data-visualization code written according to content and design specifications provided by the authors; and (iv) to format the manuscript according to the journal template. All AI-assisted output, including every citation, reported data point and figure, was independently verified by the authors against the original primary sources, fact-checked, and edited for scientific accuracy and clarity. The authors reviewed the output and take full responsibility for the accuracy, integrity and originality of the final content of this publication, consistent with MDPI’s policy on the use of generative AI in scholarly writing.

## 3. Why Exercise Capacity Remains Limited in the Pacemaker-Wearing Patient

Correcting bradycardia removes one limitation and leaves several others in place. Most of the residual exercise intolerance observed in these patients is accounted for by five mechanisms that are not mutually exclusive: an inadequate heart-rate response, the loss of atrioventricular synchrony at higher workloads, the dyssynchrony imposed by right ventricular stimulation, an altered relationship between heart rate and contractility, and the deconditioning that accompanies age and comorbidity. A standard exercise stress test cannot separate them, which is the reason for which this section precedes the discussion of the cardiopulmonary exercise test itself.

### 3.1. Chronotropic Incompetence and the Problem of Its Definition

Chronotropic incompetence is defined as the inability of the heart rate to increase appropriately with metabolic demand, but the operational criteria differ substantially between studies, and the reported prevalence differs with them. Brubaker and Kitzman, reviewing the field, described estimates ranging from 9% to 89%, attributing this dispersion mainly to the applied diagnostic thresholds [17]. The two families of criteria in use are the percentage of the age-predicted maximum heart rate, usually 80% or 85%, and the chronotropic index proposed by Wilkoff, which relates the employed heart-rate reserve to the employed metabolic reserve and therefore adjusts for resting heart rate and fitness [7].

Melzer et al. applied the Wilkoff definition to a cohort of 292 pacemaker-wearing patients investigated by cardiopulmonary exercise testing and found 125 of the 246 evaluable patients (51%) to be chronotropically incompetent, with prevalences of 42% in atrioventricular block, 56% in sinus node disease and 59% in atrial fibrillation [8]. That last figure refers to paced patients whose ventricular rate was controlled pharmacologically or by the device; it does not describe the patient with atrial fibrillation and preserved atrioventricular conduction, in whom the ventricular rate during exercise may instead rise steeply and unpredictably, a situation whose practical consequences are considered in Section 4.4. Their study also exposed a practical difficulty specific to this population: in 46 patients (16% of the cohort), the resting heart rate could not be determined at all because the intrinsic rate was lower than the lowest programmable pacing rate, so the chronotropic index could not be calculated [8]. The criteria developed for patients with an intact sinus node are therefore not directly transferable to the patient whose resting rate is imposed by a device.

The consequences are measurable and prognostically relevant. In the same cohort, maximum oxygen consumption and exercise duration were significantly reduced in chronotropically incompetent patients, while oxygen consumption at the anaerobic threshold was not different between groups, and 23% of the incompetent patients did not reach the anaerobic threshold, compared with 11% of the competent ones [8]. In the general population, an impaired heart-rate response to graded exercise was associated with an increased risk of coronary events and mortality in the Framingham Heart Study [18], and an impaired chronotropic response during exercise testing predicted mortality independently of established risk markers [11]. For this reason, Meine et al. proposed that chronotropic function be assessed at the anaerobic threshold rather than at peak effort, an approach that removes the dependence on maximal exertion in patients who frequently stop the test for non-cardiac reasons [19].

### 3.2. Loss of Atrioventricular Synchrony and Behaviour at the Upper Rate Limit

Atrial contraction contributes to ventricular filling, and the pacing mode determines whether this contribution is preserved. Nielsen et al. randomized 177 consecutive patients with sinus node disease to atrial or dual-chamber pacing and documented the echocardiographic and clinical consequences of the selected mode [2]. In a comparison of programming modes, symptoms of heart failure were more frequent with single-chamber than with dual-chamber pacing, and exercise capacity followed the same order, being best with dual-chamber devices and an active rate-responsive function and worst with single-chamber devices and the function deactivated [13].

A second and less frequently discussed problem appears at the upper end of the rate range. When the atrial rate exceeds the programmed upper rate limit, the device can no longer maintain one-to-one atrioventricular conduction, and Wenckebach behaviour or two-to-one block follows, with an abrupt fall in ventricular rate at a moment when the patient is still increasing the workload. Kindermann et al. directly addressed the question of how this limit should be defined during exercise [5]. In practice, the limit is frequently left at the value set at implantation, which may be appropriate for a sedentary patient and clearly insufficient for one entering a rehabilitation programme.

### 3.3. Ventricular Dyssynchrony Induced by Right Ventricular Pacing

Pacing from the right ventricular apex produces an activation sequence that resembles left bundle branch block, with regional differences in workload, perfusion and wall thickening; Tops et al. reviewed these mechanisms and their therapeutic implications [20]. The clinical consequences were established by two large trials. In the MOST substudy, Sweeney et al. showed that a high cumulative percentage of ventricular pacing increased the risk of heart failure hospitalization and atrial fibrillation in patients with sinus node dysfunction and normal baseline QRS duration, even when atrioventricular synchrony was preserved [21]. In the DAVID trial, Wilkoff et al. found that dual-chamber pacing offered no advantage over ventricular backup pacing in patients with an implantable defibrillator, with the excess of ventricular pacing being the plausible explanation [22].

Pacing-induced cardiomyopathy is the chronic expression of the same process. Somma et al. pooled 26 studies including 57,993 patients and confirmed both the heterogeneity of the definitions used and the association with a high ventricular pacing burden [23]; Kiehl et al. had previously described its incidence and predictors in patients with complete atrioventricular block and preserved left ventricular systolic function [24]. Conduction system pacing has emerged as the physiological alternative, with its place being set out in the 2021 European guidelines [1] and revised in detail by Glikson et al. in the 2025 consensus statement [10]. What remains poorly documented is the behaviour of the different pacing sites during effort. Almost all of the comparative evidence concerns the resting ejection fraction, and a patient may have a preserved ejection fraction at rest and nevertheless lose stroke volume when the paced rate rises.

### 3.4. The Altered Relationship Between Heart Rate and Contractility

In the normal heart, contractility increases with heart rate. In chronic heart failure, this relationship is blunted, and the heart rate above which contractility no longer increases, i.e., the critical heart rate, is reached earlier. Gierula et al. measured the three components of the force–frequency relationship non-invasively in 90 patients with chronic heart failure (mean age of 73.6 ± 8.9 years and mean left ventricular ejection fraction of 33.5 ± 10.9%) and in 15 subjects with preserved ventricular function (ejection fraction of 55.6 ± 5.3%), then compared, in a double-blind randomized crossover design, rate-response programming tailored to the critical heart rate against conventional programming; the tailored programming improved exercise time [25].

This explains an observation that would otherwise appear contradictory. Jamil et al. showed that chronotropic incompetence does not limit exercise capacity in chronic heart failure and that augmenting the heart rate by rate-adaptive pacing did not improve exercise tolerance in this population [26], a result concordant with the subgroup analysis of Kuroda et al., in which the gain in peak oxygen consumption was 1.95 mL/kg/min (95% CI: 0.66 to 3.23) in patients without heart failure and only 0.36 mL/kg/min (95% CI: −0.88 to 1.60) in those with heart failure [9]. In other words, the target is not the highest achievable heart rate but the rate beyond which the ventricle no longer converts frequency into output, and that rate differs between a patient with isolated conduction disease and one with a dilated, poorly contracting ventricle.

### 3.5. Deconditioning, Age and Comorbidity

The mechanisms described above act on a population that is old and rarely free of associated disease. In the cohort of Melzer et al., coronary artery disease, acquired valvular heart disease and previous cardiac surgery were each significantly more frequent among chronotropically incompetent patients, as was chronic treatment with digitalis, beta-blockers or amiodarone [8]. Beta-blockade deserves separate mention, since it is prescribed to most of these patients for reasons unrelated to the device and it lowers both the resting and the peak heart rate so that a chronotropic response classified as inadequate may reflect pharmacological rate control rather than intrinsic sinus node disease. Prolonged inactivity after implantation adds a peripheral component: reduced muscle mass, impaired oxygen extraction and an earlier ventilatory threshold, all of which lower peak oxygen consumption without any change in device function.

These five mechanisms produce overlapping clinical pictures, and none of them can be identified with certainty based on exercise duration, the maximum workload or the heart rate recorded at peak effort. Their separation requires measurement of gas exchange, which is the subject of the following section.

## 4. Performing and Interpreting the Cardiopulmonary Exercise Test in the Pacemaker-Wearing Patient

### 4.1. General Framework and Choice of Protocol

The performance and interpretation of cardiopulmonary exercise testing are covered by well-established documents: the ATS/ACCP statement [27], the American Heart Association clinician’s guide [6], the recommendations of the Exercise Physiology Section of the European Association for Cardiovascular Prevention and Rehabilitation for chronic heart failure [28], and the joint EACPR/AHA algorithms published in 2012 [29] and revised in the 2016 focused update [30]. None of these addresses the paced patient as a separate category, and the required additions are the subject of this section.

The protocol should be individualized so that the incremental phase lasts approximately 8 to 12 min, which is the interval over which the ventilatory threshold and the oxygen uptake efficiency are most reliably identified [29,30]. A ramp protocol is preferable to a stepwise one in this population for a reason that is specific to it: with step increments, the rate-response sensor is stimulated abruptly at each stage change, and the resulting heart-rate profile reflects the reaction time of the algorithm rather than the metabolic demand of the patient.

The choice between a cycle ergometer and treadmill is not neutral either. Accelerometer-based sensors respond to body movement and to vertical acceleration in particular, and they are therefore poorly stimulated by cycling, while sensors based on minute ventilation or on closed-loop stimulation respond to a physiological signal that is independent of the exercise modality. Leung et al. compared rate response and oxygen uptake kinetics between different sensor modes in multisensor rate-adaptive pacing and documented these differences [31], and Page et al. subsequently compared the cardiopulmonary response to exercise in recipients of dual-sensor DDDR pacemakers with that of a healthy control group [32]. The practical consequence is that a patient with an accelerometer-driven device may appear chronotropically incompetent on a cycle ergometer and adequate on a treadmill, and the sensor type must therefore be recorded together with the test result. In our own studies, we used a cycle ergometer with an adjusted Bruce protocol [13] and, subsequently, cardiopulmonary exercise testing on two occasions at 24 h intervals [14].

### 4.2. What Has to Be Recorded in Addition to the Standard Variables

Beyond maximum oxygen consumption, oxygen consumption at the ventilatory threshold determined by the V-slope method [33], the oxygen pulse and the VE/VCO_2_ slope, three additional items should be documented in every test performed in a pacemaker-wearing patient.

The first is the relationship between heart rate and oxygen consumption across the entire test, not only the heart rate reached at peak effort. This relationship is the physiological record of how the algorithm behaved under load, and it is the element that permits reprogramming to be targeted, as developed in Section 6.

The second is the resting heart rate, together with the programmed lower rate, since the chronotropic index cannot be calculated when the intrinsic rate is below the lowest programmable pacing rate. Melzer et al. were unable to determine the resting heart rate in 46 of their 292 patients, that is, in 16% of the cohort, for exactly this reason [8]. Meine et al. proposed assessment of the chronotropic response at the anaerobic threshold rather than at peak effort [19], which has the additional advantage of not depending on maximal exertion in patients who frequently interrupt the test for peripheral reasons.

The third is the device data itself. The proportion of paced and sensed beats, the pacing site, the sensor type and its programmed slope, the threshold and reaction time, the upper rate limit and any mode switch episodes occurring during the test should all be retrieved by interrogation before and after the examination. The EAPC/EHRA consensus document on cardiac rehabilitation in recipients of cardiac implantable electronic devices, published by Pedretti et al., treats device interrogation as an integral part of the assessment rather than as an optional addition [15] and a description of the sensor and of its settings is available in the review of rate-responsive technologies published by Świerżyńska et al. [34].

### 4.3. Testing with the Rate Response Deactivated, Reactivated, or Both

Two designs are possible, and they answer different questions. Melzer et al. programmed all pacemakers to non-rate-adaptive modes during testing, which allowed the native sinus node response to be evaluated and the chronotropic index to be applied to the patient rather than to the device [8]. This is the appropriate design when the question is whether chronotropic incompetence is present. When the question is, instead, what the device contributes, the patient has to be tested in the mode in which he will exercise. In our second study, we tested 33 patients on two occasions at 24 h intervals—initially in single-chamber ventricular pacing at a fixed rate of 60 bpm and subsequently in dual-chamber pacing with an exercise-adaptable rate up to 130 bpm—and the differences in maximum oxygen consumption, in oxygen consumption at the anaerobic threshold, in exercise duration and in maximum workload were all significant in favour of the second mode [14].

The handling of chronic medication before the test is a related methodological choice on which the literature is divided. One approach withdraws beta-blocker treatment 24 h before the test and resumes it immediately afterwards, with the intention of obtaining a chronotropic response as close as possible to the intrinsic one [13]. Melzer et al. took the opposite position, maintaining antiarrhythmic therapy as prescribed and arguing that testing after withdrawal represents an artificial state compared with the conditions under which the patient actually exercises [8]. The latter view is more defensible for the purpose of prescribing training. The rate response that matters clinically is the one the patient has while taking his medication and while training, and a value obtained after withdrawal describes a physiological state the patient does not occupy outside the laboratory. Withdrawal does not affect the internal validity of a comparison in which all patients are tested under the same conditions, but it does mean that the obtained absolute values do not correspond to those the patient would reach during a rehabilitation session. Where the purpose is specifically to characterise sinus node function rather than to prescribe training, withdrawal remains defensible, provided it is declared as a deliberate departure and the result is not used to set training intensity.

### 4.4. Safety and the Specific Precautions in Defibrillator Recipients

Exercise testing and exercise training are well tolerated in this population. Pandey et al. addressed safety and efficacy in patients with an implantable cardioverter–defibrillator in a meta-analysis [35], a Cochrane review of exercise-based cardiac rehabilitation in defibrillator recipients reached comparable conclusions [36], and Heidbuchel and Carré reviewed exercise and competitive sport in this group [37]. Iliou et al. described the practical organization of rehabilitation in patients with pacemakers and defibrillators [38], and the EAPC/EHRA consensus document sets out the framework currently recommended in Europe [15].

Specific to the defibrillator recipient are three precautions, each better verified before the test than during it. The lower limit of the ventricular tachycardia detection zone has to be known, and the heart rate anticipated at peak effort has to remain below it, since a rate entering the detection zone can trigger inappropriate therapy; the discrimination algorithms should be reviewed; and the mode-switch thresholds should be checked in patients with a history of atrial arrhythmia, since an atrial rate exceeding the mode-switch threshold during exercise will produce an abrupt change in ventricular response [3].

Two approaches are then defensible, and the choice depends on the patient. In most recipients, it is sufficient to know the detection limit and to conduct the test so that it is not reached, which is our routine practice. Temporary deactivation of the antitachycardia therapies for the duration of the test, with reactivation immediately afterwards, is the alternative, and we do not exclude it in two situations. The first is the case in which the patient referred precisely because of inappropriate shocks occurring during physical effort, where the purpose of the test is to reproduce the circumstances under which the therapy was delivered. The second is the case in which the patient is in atrial fibrillation, in whom the ventricular rate rises during exercise more steeply and less predictably than the sinus rate does in a patient in sinus rhythm so that the detection zone may be reached at a workload that cannot be anticipated from the resting recording. Rapidly conducted atrial fibrillation is, in fact, the commonest mechanism of inappropriate therapy: in the MADIT II analysis of Daubert et al., atrial fibrillation accounted for 81 of 184 inappropriate shock episodes, other supraventricular tachycardias including sinus tachycardia accounted for 67, and abnormal sensing accounted for 36 [39]. Whichever route is chosen, it should be recorded in the test report, and the decision belongs jointly to the physician performing the test and to the electrophysiologist following the device.

### 4.5. Errors of Interpretation Specific to the Paced Patient

Several findings are regularly misread, three of them often enough to be worth setting out.

An abrupt fall in ventricular rate in the final minutes of the test, with the patient still increasing the workload, is frequently interpreted as chronotropic incompetence or as fatigue when it, in fact, represents the upper rate behaviour of the device, Wenckebach conduction or a two-to-one block appearing once the atrial rate exceeds the programmed limit [5]. The distinction is straightforward if the upper rate limit is known before the test and impossible if it is not.

A heart rate that rises apparently normally does not establish that the chronotropic response is appropriate because in a paced patient, that rate may have been generated by the sensor rather than by the sinus node. The two situations are separated by the proportion of paced beats, which is why interrogation matters.

Finally, the electrocardiographic tracing recorded during atrial or ventricular paced beats does not allow for reliable assessment of repolarization so that ischaemic changes and some arrhythmias cannot be excluded on the exercise electrocardiogram alone in these patients. This limitation was one of the reasons for which we considered cardiopulmonary exercise testing preferable to the classical exercise test in this population, since the gas exchange variables remain interpretable when electrocardiogram evidence is not.

## 5. The Cardiopulmonary Variables That Matter in the Pacemaker-Wearing Patient

The variables produced by a cardiopulmonary exercise test are the same in every patient. What changes in the paced patient is the weight that should be given to each of them because one of the determinants of the response, i.e., the heart rate, is partly imposed by a device rather than generated by the sinus node. The variables discussed in this section were selected on that basis: peak oxygen consumption, the anaerobic threshold, the oxygen pulse, ventilatory efficiency and the heart rate-to-oxygen consumption relationship are the measurements whose interpretation is altered by pacing or that serve as a rate-independent reference against which the rate-dependent variables can be judged. Measurements that the presence of a pacemaker does not specifically modify, such as the breathing reserve or the respiratory exchange ratio, retain their conventional meaning and are not discussed separately.

### 5.1. Peak Oxygen Consumption

Peak oxygen consumption remains the reference measure of exercise capacity and the variable with the longest prognostic history since Mancini et al. established its value for the timing of cardiac transplantation in ambulatory patients with heart failure [40]. It is also the variable that responds to reprogramming: it rises when a device is changed from a single-chamber, fixed-rate mode to a dual-chamber, rate-adaptive mode [14], and in the meta-analysis of Kuroda et al., the pooled gain associated with activating the rate-adaptive mode was 1.35 mL/kg/min (95% CI: 0.47 to 2.23) [9].

Two cautions apply in this population. Peak oxygen consumption is effort-dependent, and it is interpretable as a maximal value only when the respiratory exchange ratio confirms that the effort was maximal; in patients who stop for orthopaedic or peripheral reasons, which is frequent at the ages concerned, the obtained value is a symptom-limited one and should be reported as such. Second, low peak oxygen consumption does not identify its own cause. It is the same whether the limitation arises from an inadequate rate response, from ventricular dyssynchrony, or from deconditioning, which is precisely why the remaining variables are needed.

### 5.2. The Anaerobic Threshold and the Meaning of Not Reaching It

Oxygen consumption at the anaerobic threshold, determined by the V-slope method [33], is effort-independent and therefore particularly suited to a population that rarely performs a truly maximal test. It also increases with reprogramming, and in at least one series, the change reached a lower *p* value than the change in peak oxygen consumption [14].

The observation of Melzer et al. deserves more attention than it has received: 23% of chronotropically incompetent patients did not reach the anaerobic threshold at all, compared with 11% of the chronotropically competent ones (*p* = 0.023), while oxygen consumption at the threshold did not differ between the two groups when it could be measured [8]. The failure to reach the threshold is therefore, itself, a finding rather than a technical failure, and it carries prognostic weight in heart failure, as Agostoni et al. showed for the indeterminable anaerobic threshold in the MECKI cohort [41]. In a paced patient, an inability to reach the threshold, together with a heart rate that stopped rising early, points towards the device rather than towards the periphery.

### 5.3. The Oxygen Pulse

The oxygen pulse, i.e., the ratio of oxygen consumption to heart rate, is conventionally read as a surrogate for stroke volume multiplied by the arteriovenous oxygen difference, and a plateau during incremental exercise is taken to indicate a stroke volume that can no longer increase.

This interpretation requires modification when the heart rate is device-determined, and the modification runs in the opposite direction of the one clinicians expect. If the upper rate limit is reached early or if the sensor slope is set too low, the denominator of the ratio stops increasing while oxygen consumption continues to rise, and the oxygen pulse will therefore continue to climb or will climb more steeply at the end of the test. A rising oxygen pulse in this setting is therefore not necessarily reassuring: it may be the arithmetic consequence of a heart rate that has been artificially capped rather than a sign of preserved stroke volume. This interpretation follows from the definition of the ratio rather than from validation studies specific to paced patients and should be applied with corresponding caution. Conversely, an oxygen pulse that plateaus in a patient whose paced rate is still rising retains its usual meaning and indicates a stroke volume limitation, which, in this population, raises the question of pacing-induced dyssynchrony. The oxygen pulse should therefore never be read without the heart-rate curve beside it.

### 5.4. Ventilatory Efficiency

The VE/VCO_2_ slope is effort-independent; reproducible; and, in several comparisons, at least as strong a discriminator of prognosis in heart failure as peak oxygen consumption [42,43], with its role having been reassessed by Corrà et al. in the context of contemporary heart failure treatment [44]. The oxygen uptake efficiency slope, as described by Baba et al. [45] and further characterized by Hollenberg and Tager as an index obtainable from submaximal exercise [46], is useful for the same reason and in the same patients. The usefulness of the oxygen uptake efficiency slope extends beyond cardiac patients: it has been characterized as a transferrable submaximal index of cardiorespiratory response in endurance athletes [47], which supports its use as a mode-independent reference in the paced patient.

Neither variable is influenced directly by the pacing mode, and that is exactly what makes them valuable here. They provide a stable reference against which the rate-dependent variables can be judged, and they allow a repeat test performed after reprogramming to be interpreted with some confidence that any change in peak oxygen consumption reflects the intervention rather than a difference in effort between the two occasions. A related index, the oxygen uptake efficiency plateau, has been proposed as a stable measure transferable between clinical and healthy, well-trained populations [48]; its behaviour in pacemaker recipients has not been examined and remains an under-investigated area.

### 5.5. The Heart Rate-to-Oxygen Consumption Relationship

This is the variable that should be given the central place in the paced patient, and it is the one most often omitted from reports.

The chronotropic index of Wilkoff relates the heart-rate reserve used to the metabolic reserve used [7], but it requires a resting heart rate, which cannot be determined when the intrinsic rate lies below the programmed lower rate, a situation encountered in 16% of the cohort of Melzer et al. [8]. Two alternatives avoid this difficulty. Kindermann et al., working on the definition of the optimum upper rate limit, characterized chronotropic incompetence upon cardiopulmonary testing as an increase in heart rate of less than 2 bpm for each mL/kg/min of oxygen consumption [5]. Meine et al. proposed assessment of the chronotropic response at the anaerobic threshold, which removes the dependence on maximal effort [19]. The kinetics of the relationship, rather than its slope alone, were examined by Kay et al. during constant-workload exercise [49].

Read as a curve across the whole test, this relationship distinguishes patterns that a single peak heart rate cannot. A slope that is uniformly flat from the beginning suggests either an inactive sensor or one whose gain is set too low. A slope that is normal at low workloads and flattens abruptly in the last minutes suggests the upper rate limit rather than chronotropic incompetence. A delay between the onset of exercise and the first rise in rate points to the reaction time of the sensor. A rate that rises, then falls abruptly suggests Wenckebach behaviour or a mode switch. Each of these patterns has a different programming consequence. They are shown schematically in Figure 1, and their programming consequences are developed in Section 6.

### 5.6. A Proposed Minimum Reporting Set

Table 1 lists what we consider the minimum that should appear in the report of a cardiopulmonary exercise test performed in a pacemaker-wearing patient. The left-hand column contains the standard variables, which require no justification; the right-hand column contains the additions specific to this population.

## 6. From the Cardiopulmonary Exercise Test to Device Programming

### 6.1. What the Test Can and Cannot Dictate

A cardiopulmonary exercise test does not prescribe a set of pacemaker parameters. What it provides is a description of how the device behaved while the patient was working, and that description narrows the programming decision from a general preference to a specific correction. The distinction matters because the alternative currently in use is not a different method but the absence of one: in most centres, the settings applied at implantation are never revisited unless the patient becomes symptomatic.

The reasoning proceeds in three steps. The heart rate-to-oxygen consumption curve is inspected for the pattern it shows; the pattern is attributed to a component of the programming, and the correction is verified by a repeat test rather than assumed. Section 5.5 describes four patterns, and Table 2 sets out the programming consequence of each. The sequence, as a whole, is summarized in Figure 2.

### 6.2. Choosing and Calibrating the Sensor

Rate-adaptive pacing is delivered by sensors that differ in the signal they detect and therefore in the situations in which they perform well. Accelerometers respond to body movement, react quickly and are insensitive to non-exercise physiological demand; minute ventilation sensors follow a metabolic signal and therefore track workload more faithfully, at the cost of a slower onset; closed-loop stimulation derives the rate from changes in intracardiac impedance related to contractility and therefore responds to emotional as well as physical demand; and blended sensors combine an accelerometer for the onset with a metabolic sensor for the plateau. Świerżyńska et al. have reviewed these technologies and their applications in detail [34], and the differences in rate response and in oxygen uptake kinetics between sensor modes were quantified by Leung et al. [31] and compared against a healthy control group by Page et al. [32].

Three programmable parameters govern the behaviour of any of them. The activity threshold determines the level of the input signal at which the rate begins to rise, and a threshold set too high produces a heart rate that remains at the lower rate during the first minutes of exercise. The slope, or gain, determines how much the rate rises for a given input, and it is the parameter that corresponds most directly to the heart rate-to-oxygen consumption relationship measured in the test. The reaction and recovery times determine how quickly the rate follows the onset and the cessation of effort. In our experience, these are the three settings that are most often left at nominal values and that most often require correction.

### 6.3. The Upper Rate Limit and the Atrioventricular Delay

The upper rate limit is the parameter most likely to be inappropriate in a patient entering a rehabilitation programme, since the value chosen at implantation reflects the activity level of a patient who has just been implanted. When the atrial rate exceeds it, atrioventricular synchrony is lost through Wenckebach behaviour or two-to-one block, and the ventricular rate falls abruptly while the workload is still increasing. Kindermann et al. examined how this limit should be defined during exercise [5], and the test itself identifies the problem unambiguously because the fall occurs at a reproducible rate that corresponds to the programmed value.

The atrioventricular delay is a separate consideration and one that is almost always programmed for rest. A delay that produces adequate filling at 60 bpm may be too long at 110 bpm, when diastole is short and atrial contraction risks encroaching on the preceding ventricular systole. Rate-adaptive shortening of the atrioventricular delay exists in most contemporary devices and is frequently not activated.

### 6.4. When More Rate Is Not Better

The temptation, once a rate response is shown to be inadequate, is to increase it as far as the device permits. Three lines of evidence indicate that this is wrong in an identifiable subgroup.

Jamil et al. showed that chronotropic incompetence does not limit exercise capacity in chronic heart failure and that augmenting the heart rate by rate-adaptive pacing did not improve exercise tolerance in that population [26]. Gierula et al. provided the mechanism: in the failing ventricle, the force–frequency relationship is blunted and, above a critical heart-rate contractility, no longer increases so that programming tailored to that critical rate improved exercise time, whereas conventional programming did not [25]. The effect was subsequently shown to persist: in a single-centre, double-blind, randomized, parallel-group trial in patients with stable symptomatic heart failure with reduced ejection fraction on optimal guideline-directed therapy carrying a resynchronization or defibrillator device, the same group tailored rate-response programming to the individually determined critical heart rate and reported improved exercise tolerance six months later [50]. The approach has not been accepted without objection: the trial drew a published letter of critique, to which the authors replied [51,52]. Evidence on rate augmentation applied without individual titration points the other way. Proff et al. randomized patients with chronic heart failure and severe chronotropic incompetence to closed-loop stimulation in a crossover design and, despite raising peak heart rate by 13 ± 9 bpm, found no change in gas exchange variables, quality of life, activity status or six-minute walk distance [53]. Reddy et al. then tested the strategy prospectively in the RAPID-HF trial, implanting pacemakers in 29 patients with heart failure with preserved ejection fraction and chronotropic incompetence and comparing rate-adaptive atrial pacing on and off in a double-blind crossover design: exercise heart rate increased, while exercise capacity and health status did not; cardiac output did not rise because stroke volume fell; and pacemaker implantation was associated with adverse events [54]. The subgroup analysis of Kuroda et al. points in the same direction, with a gain in peak oxygen consumption of 1.95 mL/kg/min (95% CI: 0.66 to 3.23) in patients without heart failure and 0.36 mL/kg/min (95% CI: −0.88 to 1.60) in those with heart failure [9]. The same divergence appears in the meta-analysis by Kuroda et al.: pooling 12 randomized trials in 1199 patients, activation of the rate-adaptive mode increased peak oxygen consumption by 1.95 mL/kg/min (95% CI: 0.66 to 3.23) in patients without heart failure but by only 0.36 mL/kg/min (95% CI: −0.88 to 1.60) in those with heart failure [9].

The objective of reprogramming is therefore not the highest rate the device can deliver but the highest rate at which the ventricle still converts frequency into output, and this ceiling has to be established for the individual patient rather than assumed from the age-predicted maximum. In a patient with isolated conduction disease and a normal ventricle, the two values may coincide; in a patient with a dilated, poorly contracting ventricle, they clearly do not.

### 6.5. Beyond Rate: Pacing Site and the Burden of Ventricular Pacing

Rate is not the only programmable determinant of exercise capacity. The proportion of beats delivered by the ventricular lead has consequences that are independent of frequency, as shown by Sweeney et al. in the MOST substudy [21] and by Wilkoff et al. in the DAVID trial [22], and the chronic expression of a high pacing burden is the pacing-induced cardiomyopathy characterized by Somma et al. [23], with the underlying mechanisms reviewed by Tops et al. [20]. Algorithms that promote intrinsic atrioventricular conduction should therefore be activated whenever conduction permits, and this decision is made in the same consultation as those concerning the rate-response settings.

Where ventricular pacing is unavoidable, the site becomes relevant. Conduction system pacing preserves a more physiological activation sequence, with its indications being set out in the 2021 European guidelines [1] and revised by Glikson et al. in the 2025 consensus statement [10]. The evidence supporting it concerns mainly the resting ejection fraction, and comparative data on exercise performance between right ventricular and conduction system pacing are, to the best of our knowledge, sparse. This is one of the gaps identified in Section 9.

### 6.6. Evidence That Reprogramming Changes the Test Result

The proposition underlying this section is that reprogramming produces a measurable change, and it has been tested directly.

Comparisons of programming modes have shown that exercise capacity is highest with dual-chamber pacing and an active rate-responsive function and lowest with single-chamber pacing and the function deactivated, with activation of the function being associated with significant gains in exercise duration, maximum workload and metabolic equivalents [13].

The change can be demonstrated within 24 h: when the same patients are tested on consecutive days in a single-chamber, fixed-rate mode, then in a dual-chamber, rate-adaptive mode, maximum oxygen consumption, oxygen consumption at the anaerobic threshold, exercise duration and maximum workload are all significantly higher in the rate-adaptive mode [14]. The effect therefore does not require a training period to become apparent, which has a direct practical consequence: the settings should be corrected before the rehabilitation programme begins, not at its end.

The approach has also been reported at the level of the individual patient. Hsieh et al. described a 70-year-old lifelong runner with a pacemaker implanted for sinus node dysfunction who presented with exercise intolerance and dyspnoea on exertion, in whom a maximal cardiopulmonary exercise test performed simultaneously with device interrogation produced an optimized settings profile that resolved his symptoms [55]. The authors state plainly that nominal rate modulation settings do not reproduce physiological exercise well. A single case establishes nothing on its own, but it documents the entire sequence, from complaint to testing, reprogramming and symptomatic result, which the group studies cited above do not.

Other groups have addressed adjacent questions. Palmisano et al. compared fixed-rate with rate-responsive pacing in patients with permanent atrial fibrillation treated by atrioventricular junction ablation and biventricular pacing in the RESPONSIBLE study [56], Abi-Samra et al. examined the effect of rate-adaptive pacing on performance during activities of daily living in elderly patients in the CLEAR study [57], and Serova et al. reported an algorithm for optimizing rate-adaptive pacing in patients with heart failure with preserved ejection fraction [58].

## 7. Prescribing Exercise Training from the Cardiopulmonary Exercise Test

### 7.1. Why Heart-Rate-Based Prescription Fails in the Paced Patient

Training intensity in cardiac rehabilitation is conventionally prescribed as a percentage of the maximum heart rate or of the heart-rate reserve. Both formulations rest on three assumptions, and all three fail when the rate is generated by a device.

The first is that a resting heart rate can be measured. In a paced patient, the rate recorded at rest is frequently the programmed lower rate rather than the intrinsic one, and Melzer et al. could not determine the resting rate at all in 46 of 292 patients, that is, in 16% of their cohort, because the intrinsic rate lay below the lowest programmable value [8]. The heart-rate reserve calculated from such a value is not a physiological quantity.

The second is that the maximum heart rate is predictable from age. In a paced patient, the ceiling is set by the programmed upper rate limit or by the maximum sensor-driven rate, and these are programmed values that bear no relation to the age-predicted maximum. A patient whose upper rate limit is 120 bpm cannot reach 85% of an age-predicted maximum of 150 bpm, and describing him as having failed to achieve target intensity is a description of the device, not of the patient.

The third is that the relationship between heart rate and oxygen consumption is monotonic and reasonably uniform. In the paced patient, this relationship is determined by the sensor slope, which means that the same nominal percentage of heart-rate reserve corresponds to different metabolic intensities in two patients with different programming and in the same patient before and after reprogramming.

Beta-blockade compounds all three, since it is prescribed to most of these patients for reasons unrelated to the device.

The convention nevertheless remains in use in published trials, as well as in clinical practice, and this reflects the absence of an established alternative rather than any oversight. Squeo et al., for instance, reported a well-conducted training programme in 30 recipients of implantable cardiac devices, 25 of whom carried a pacemaker, which produced significant improvement in peak oxygen consumption, from 15 ± 4 to 17 ± 4 mL/kg/min, and in maximum workload, from 87 ± 30 to 108 ± 37 W, with aerobic intensity prescribed as a percentage of the heart-rate reserve [59]. Their results establish that exercise training is effective and safe in this population. Our point concerns only the metric: in a paced patient, the heart-rate reserve is not the quantity it is assumed to be, and an intensity domain defined from it is therefore difficult to reproduce between patients or in the same patient after reprogramming.

### 7.2. Threshold-Based Prescription

The alternative was set out by Mezzani et al. in the joint position statement of the European Association for Cardiovascular Prevention and Rehabilitation, the American Association of Cardiovascular and Pulmonary Rehabilitation and the Canadian Association of Cardiac Rehabilitation, which proposed a threshold-based rather than a range-based approach, with the intensity domains being delimited by the first and second ventilatory thresholds rather than by percentages of peak capacity [60]. The subsequent position statement of the Secondary Prevention and Rehabilitation Section of the European Association of Preventive Cardiology revisited the question and confirmed that intensity is determined more accurately by reference to the ventilatory thresholds than as a percentage of peak exercise capacity [61]. The standards for the functional evaluation of cardiac patients published by the Exercise Physiology Section of the same association describe the measurements involved [62].

The reason this matters here is specific rather than general. Ventilatory thresholds are measured directly from gas exchange and are not derived from the heart rate, which means they survive the device dependence that invalidates the heart-rate formulations intact. A patient trained at a workload corresponding to his first ventilatory threshold is trained at that metabolic intensity whether his rate at that moment was generated by his sinus node, by an accelerometer or by a fixed lower rate.

### 7.3. Absolute Workload and Perceived Exertion

Two practical anchors follow from the same test. The workload in watts corresponding to the ventilatory threshold is directly transferable to a cycle ergometer in the rehabilitation gymnasium, and it is the simplest prescription to apply and to verify. Ratings of perceived exertion on the Borg scale provide the second anchor and are particularly useful in this population, since they are unaffected by the pacing mode, are available in the absence of any monitoring, and allow the patient to regulate their effort during the sessions performed at home. The general standards for exercise testing and training set out by Fletcher et al. remain applicable [63].

We would go further and suggest that in a paced patient, the heart rate should be monitored during training for safety and for the detection of upper rate behaviour or mode switch but that ventilatory thresholds and workload, when they are available, are preferred for setting intensity. Heart=rate targets retain a role only in selected situations, for instance, when gas-exchange data cannot be obtained or when the paced heart rate can be shown to track metabolic demand in a stable and predictable manner. This reprioritization of the variable, rather than its abandonment, is the practical conclusion of this review.

### 7.4. Continuous and Interval Training

The position statement of the European Association of Preventive Cardiology considers moderate-intensity continuous training the most feasible modality for most patients referred to rehabilitation, with high-intensity interval training reserved for selected patients and specific objectives such as increasing peak oxygen consumption [61].

An additional consideration applies to the paced patient, and it follows from the programmable parameters themselves rather than from a general physiological argument. The reaction time governs how quickly the sensor-driven rate rises once the activity threshold has been exceeded, and the recovery time governs how quickly it falls again, each operating by requiring the device to pace for a defined number of cycles at a given rate before moving to the next [34]. Both are programmed in tens of seconds to minutes: in the case of cardiopulmonary-guided optimization reported by Hsieh et al., the accelerometer reaction time was set at 30 s, and the recovery time was set at 2 min [55]. An interval protocol built on work periods of one to four minutes separated by recovery periods of comparable length therefore operates on the same timescale as the algorithm that has to follow it, and with a recovery time of 2 min, the paced rate cannot return towards the lower rate within a one-minute recovery period, whatever the patient does. The delivered profile is a smoothed version of the prescribed profile. Where interval training is intended, the reaction and recovery times should be reviewed before the programme begins, and the heart-rate profile recorded during a trial session shows directly how far the delivered profile departs from the intended one.

### 7.5. What Has Been Demonstrated in Device Recipients and What Has Not

Exercise training in this population is effective and safe. The FIDE project documented improvement in peak oxygen consumption, workload and muscle strength after a two-month programme [59], Greco et al. addressed cardiac rehabilitation specifically in recipients of rate-responsive pacemakers [4], Pandey et al. [35] and the Cochrane review [36] established safety in defibrillator recipients; the consensus document of Pedretti et al. provides the European framework [15], and Di et al. summarized the evidence on risk stratification for exercise rehabilitation in recipients of cardiac implantable electronic devices [64].

What has not been demonstrated is the proposition that this review advances. We are not aware of a trial comparing training prescribed from cardiopulmonary thresholds against training prescribed from heart-rate percentages in pacemaker recipients or of a trial in which device reprogramming preceded a rehabilitation programme and the two were evaluated together. The rationale for both is set out in Section 6 and Section 7, and the absence of direct evidence should be stated plainly rather than implied, which is done in Section 9.

## 8. Special Populations

The framework described in Section 6 and Section 7 applies to a patient with a conventional bradycardia device. It requires modification in five groups, and in three of them, what changes is the objective rather than the method.

### 8.1. Recipients of Cardiac Resynchronization Therapy

Cardiac resynchronization improves functional capacity and outcome in selected patients with heart failure [65], and recipients are referred to rehabilitation in increasing numbers. Ambrosetti et al. wrote the practical guide for this population [66]; Belardinelli et al. demonstrated that moderate exercise training improves functional capacity, quality of life and endothelium-dependent vasodilation in patients with defibrillators and resynchronization devices [67]; and the rehabilitation of the resynchronized patient was described by Zdrenghea et al. [68].

The variable that governs the result is the percentage of effective biventricular capture, a variable that behaves differently during exercise than at rest. Capture can be lost under effort through several mechanisms: an atrial rate exceeding the upper tracking limit, fusion with intrinsic conduction as the atrioventricular interval shortens, or an atrial arrhythmia triggering a mode switch [3]. A patient whose device reports 98% biventricular pacing upon interrogation may be pacing far less than at the intensity at which he trains, and the cardiopulmonary exercise test, performed with the device interrogated afterwards, is one of the few routine methods able to demonstrate this directly. Where resynchronization has been delivered adequately at rest and the patient still fails to improve, this is the first question to ask.

Rate response is relevant in this group, as well as capture. Tse et al. examined the incremental benefit of rate-adaptive pacing with respect to exercise performance in patients receiving cardiac resynchronization therapy and found that activating the rate-adaptive mode added to the improvement obtained from resynchronization alone [69]. The two adjustments are complementary rather than alternative: restoring synchrony does not remove the need to verify that the rate rises appropriately with workload.

### 8.2. Permanent Atrial Fibrillation with Single-Chamber Ventricular Pacing

This group deserves separate treatment because in these patients, the sensor is not one contributor to the rate response but the only one. There is no atrial activity to track, so the entire adaptation of rate to workload passes through the algorithm, and the consequences of a poorly calibrated sensor are correspondingly greater.

In a comparison of programming modes, single-chamber devices with an active rate-responsive function achieved an exercise capacity that did not differ significantly from that of dual-chamber devices with the function deactivated, whereas single-chamber devices with the function deactivated performed worst [13]. The practical conclusion is the following: activating a single function of the device confers a benefit comparable to preserving the contribution of atrial contraction so that a patient in permanent atrial fibrillation with a single-chamber pacemaker and an active rate-responsive function can attain the exercise capacity of a patient with preserved atrioventricular synchrony. Given that the function is not activated by default in all device types, this is a correction that costs nothing.

Palmisano et al. examined the related situation of patients with permanent, refractory atrial fibrillation and left ventricular dysfunction treated by atrioventricular junction ablation and biventricular pacing, comparing fixed-rate with rate-responsive pacing in the RESPONSIBLE study [56]. The broader principles of cardiac rehabilitation in patients with atrial fibrillation were recently reviewed in [70].

### 8.3. Pacing-Induced Cardiomyopathy and Conduction System Pacing

Where a high ventricular pacing burden has produced a fall in the ejection fraction, the objective of the assessment changes. The question is no longer how to improve the rate response but whether the pacing strategy, itself, should be revised, and the exercise test contributes by documenting the functional cost. Somma et al. characterized the prevalence and the risk factors of pacing-induced cardiomyopathy [23], Kiehl et al. investigated its incidence in patients with complete atrioventricular block and preserved systolic function [24], and Tops et al. explored the mechanisms [20]. The indications for conduction system pacing are set out in the 2021 European guidelines [1] and in the 2025 consensus statement of Glikson et al. [10], and comparative outcome data continue to accumulate, as in the registry-based comparison of left bundle branch area pacing with conventional right ventricular pacing reported by Bertini et al. [71].

The gap is specific and worth stating: the comparative evidence concerns predominantly the resting ejection fraction, and we found no adequately powered comparison of exercise capacity, measured by gas exchange, between right ventricular and conduction system pacing. A patient may have a preserved ejection fraction at rest and lose stroke volume as the paced rate rises, which is precisely the situation a cardiopulmonary test detects and an echocardiogram at rest does not.

### 8.4. Defibrillator Recipients

The safety of exercise training in this group is established [35,36], the position on sport and exercise was reviewed by Heidbuchel and Carré [37], the practical organization was described by Iliou et al. [38] and by Di et al. [64], and the European framework is that of the consensus document of Pedretti et al. [15]. The precautions relating to the ventricular tachycardia detection zone are described in Section 4.4.

One consequence of those precautions bears on programming rather than on testing, and it is a genuine conflict between two functions of the same device. When the exercise test shows that the upper rate limit is reached prematurely, the correction proposed in Table 2 is to raise it. In a defibrillator recipient, raising it moves the maximum rate achievable during exercise closer to the lower boundary of the detection zone, and the two settings therefore have to be adjusted together rather than separately. Where the margin is insufficient, the alternatives are to accept a lower upper rate limit and to compensate through the training prescription or to review the detection zone, itself, with the electrophysiologist. We did not identify a graded recommendation addressing this trade-off in the consulted documents, and in our practice, the decision is taken jointly with the electrophysiologist based on the device data.

### 8.5. The Elderly Patient with Heart Failure and Preserved Ejection Fraction

This is the population in which the rule established in Section 6.4 matters most and in which the intuitive intervention is most likely to be wrong. Chronotropic incompetence is frequent here, and the temptation to correct it is correspondingly strong. Reddy et al. tested that intervention prospectively in the RAPID-HF trial and found that atrial pacing raised the exercise heart rate without improving exercise capacity or health status because cardiac output did not increase, stroke volume having fallen as the rate rose, while pacemaker implantation, itself, carried adverse events [54]. The mechanism is the one described by Gierula et al. for a failing ventricle [25] and reviewed for chronotropic incompetence in general by Brubaker and Kitzman [17]. Serova et al. proposed an optimization algorithm specifically for this population [58].

The conclusion for practice is restrictive rather than expansive. In an elderly patient with preserved ejection fraction, a demonstrated chronotropic incompetence is not, by itself, an indication to increase the rate, and where a device is already present, the rate response should be titrated against the measured oxygen consumption rather than against the age-predicted maximum. The peripheral component of the limitation, which is substantial at these ages, responds to training, and the training prescription is therefore the intervention with the better evidence behind it.

## 9. Limitations of the Current Evidence and Directions for Research

### 9.1. What Can Be Considered Established

Evidence of reasonable quality supports three propositions. Chronotropic incompetence is frequent in pacemaker recipients and is associated with reduced maximum oxygen consumption and shorter exercise duration [8]. Activating and calibrating the rate-responsive function improves exercise capacity in patients without heart failure, with a pooled gain in peak oxygen consumption of 1.35 mL/kg/min across 12 randomized trials that is larger in the subgroup free of heart failure [9], and the effect is measurable within 24 h of reprogramming [14]. Exercise training is effective and safe in recipients of pacemakers and defibrillators [35,36,59], and a European framework for delivering it exists [15].

### 9.2. What Is Not Established

The central proposition of this review has not been tested directly. We are not aware of a randomized comparison of training intensity prescribed based on cardiopulmonary thresholds against training prescribed on the basis of the percentage of heart-rate reserve in pacemaker recipients, and the argument advanced in Section 7 therefore rests on the invalidity of the assumptions underlying the heart-rate method rather than on a demonstration that the alternative performs better. That distinction should be preserved in the wording of the Conclusions.

Furthermore, the sequence proposed in Table 3 has not been evaluated as a whole. Reprogramming has been shown to change the test result [13,14,25,56], and training has been shown to improve exercise capacity [59], but no study has combined the two in the proposed order, with reprogramming preceding the rehabilitation programme and the effects of each separately identifiable.

The comparison between right ventricular and conduction system pacing rests almost entirely on the resting ejection fraction and on clinical outcomes [1,10,71], and we found no adequately powered comparison of exercise capacity measured by gas exchange between the two strategies.

The definitions of chronotropic incompetence currently in use were developed in populations with an intact sinus node. The chronotropic index requires a resting heart rate that cannot be determined in a substantial minority of paced patients [8], and the alternatives proposed by Kindermann et al. [5] and by Meine et al. [19] have not been validated prospectively in this population against a clinical outcome.

Finally, the interaction between the upper rate limit and the ventricular tachycardia detection zone in defibrillator recipients, as described in Section 8.4, is not covered by published guidance.

### 9.3. Recurring Methodological Problems

Across the literature, four problems recur and limit what can be concluded from it.

The definitions of chronotropic incompetence differ between studies to the point that reported prevalence ranges from 9% to 89% [17], which makes prevalence figures and treatment effects difficult to compare across series.

Sample sizes are small. Most of the studies comparing programming modes enrolled fewer than 60 patients and returned discordant results [13].

Device settings are rarely reported in rehabilitation studies, and rehabilitation outcomes are rarely reported in electrophysiological studies. A trial can therefore show that training improves peak oxygen consumption in device recipients without allowing the reader to determine whether the devices were programmed appropriately or whether part of the effect attributed to training could have been obtained by reprogramming alone.

Exercise modality and sensor type are frequently not stated together, although the response of an accelerometer differs substantially between cycle ergometry and treadmill walking [31,32].

The minimum reporting set proposed in Table 1 is intended as a partial remedy for the third and fourth of these. A further limitation is that only publications in English were considered; a scoping check carried out during revision did not identify a substantial body of original non-English studies on cardiopulmonary exercise testing in device recipients, but the possibility that relevant work in other languages was missed cannot be excluded.

### 9.4. A Research Agenda

The questions this review has had to leave open would be answered by four studies, listed here in ascending order of difficulty.

A prospective series applying cardiopulmonary exercise testing before and after targeted reprogramming, in a cohort large enough for the patterns described in Table 2 to be counted, would establish how frequently each is encountered and how many are correctable. This extends the design of existing single-centre work [14] and is, itself, feasible in a single centre.

A randomized comparison of training prescribed based on the first ventilatory threshold against training prescribed on the basis of a percentage of heart-rate reserve, in pacemaker recipients entering a rehabilitation programme, with peak oxygen consumption at the end of the programme as the primary endpoint, would test the proposition of Section 7 directly.

A factorial design separating the effect of reprogramming from the effect of training, with the four combinations of reprogramming and training present or absent, would establish whether the two are additive and would answer the practical question of sequencing.

A comparison of exercise capacity measured by gas exchange between right ventricular and conduction system pacing, ideally as a substudy of an existing registry or randomized comparison, would fill the gap identified in Section 8.3 without requiring a dedicated trial.

### 9.5. Emerging Directions

Contemporary devices record activity; heart-rate histograms; and, in some models, thoracic impedance, and these data offer an objective measure of what the patient does between rehabilitation sessions rather than what he reports. Abi-Samra et al. used device data in this way during activities of daily living [57]. Whether device-derived activity can serve as an outcome measure in rehabilitation trials and how it relates to the change in peak oxygen consumption are unresolved.

Automated interpretation of cardiopulmonary exercise tests is developing, and the paced patient is a plausible early application, since the patterns described in Section 5.5 are recognizable from the shape of a curve and the corresponding programming actions are finite in number. We are not aware of a published algorithm addressing this specific task.

Sensor technology continues to evolve towards blended and closed-loop designs [34], and leadless devices raise the question of how a sensor housed entirely within the ventricle behaves during exercise, on which we found no cardiopulmonary data.

## 10. Conclusions

The pacemaker determines a substantial and programmable part of the exercise response, chronotropic incompetence is present in about half of pacemaker recipients, and the rate-responsive function that exists to correct it is frequently left at the settings applied in the implantation laboratory. Cardiopulmonary exercise testing has particular value here because, read as the relationship between heart rate and oxygen consumption across the whole test rather than as a peak value, it identifies which component of the programming is responsible for the limitation—an inadequate sensor slope, a threshold set too high, a reaction time out of step with the onset of effort or an upper rate limit reached prematurely—each of which has a different correction.

Because reprogramming changes the result of the test, the settings should be corrected before the rehabilitation programme begins rather than reviewed at its end. The objective of that correction is not the highest rate the device can deliver but the individual ceiling above which the ventricle no longer converts frequency into output, established by measurement rather than predicted from age; in patients with heart failure, raising the rate beyond that ceiling produces no gain and may cause harm. For the prescription of training, the ventilatory threshold, the corresponding workload in watts and perceived exertion are preferred to heart rate, which, in the paced patient, is best reserved for safety and for the detection of upper-rate or mode-switch behaviour.

None of this has been tested as a whole, and the sequence proposed here is a framework derived from established mechanisms rather than a validated algorithm. What can be said with confidence is narrower and still useful: the device should be interrogated before the test, the settings should be reported with the result, and the rate response should be verified rather than assumed.

## Figures and Tables

**Figure 1:**
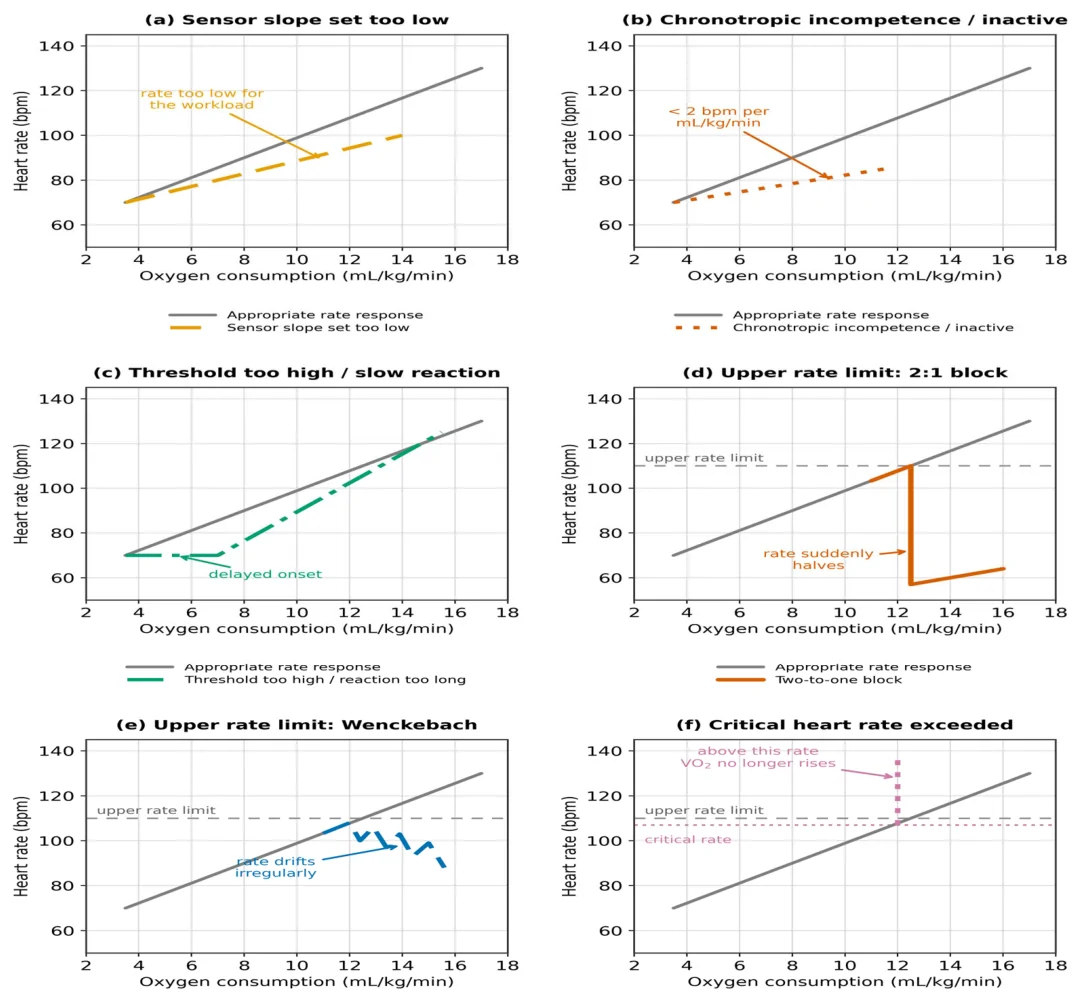
Patterns of the heart rate-to-oxygen consumption relationship in the pacemaker-wearing patient. Schematic representation, not derived from individual patient data; the patterns are conceptual illustrations intended to aid in pattern recognition and have not been validated as diagnostic phenotypes; in every panel, the grey line is the normal, appropriate rate response, shown for reference, against which the abnormal pattern (in colour) is compared. (**a**) Sensor slope set too low: the heart rate rises throughout the test but with a gradient that is too shallow, so at any given oxygen consumption, the rate is lower than it should be. (**b**) Chronotropic incompetence or an inactive rate response: the heart rate barely rises with oxygen consumption (here, a slope below 2 bpm per mL/kg/min), and peak effort is reached at a low workload. (**c**) Activity threshold set too high or reaction time too long: the heart rate stays at the lower rate during the first minutes of exercise and only climbs once effort is already substantial. (**d**) Two-to-one block: once the sensor-driven rate reaches the programmed upper rate limit, the pacemaker suddenly stops tracking one beat in two, so the ventricular rate abruptly halves before resuming its climb. (**e**) Wenckebach upper-rate behaviour: at the upper rate limit, the rate is withheld progressively rather than abruptly, so instead of a clean halving, the rate drifts down and wanders irregularly. (**f**) Critical heart rate exceeded: below a critical rate, the heart rate and oxygen consumption rise together along the reference, but once that rate is passed, the ventricle no longer converts additional frequency into additional output, so the heart rate keeps climbing while oxygen consumption no longer rises. bpm, beats per minute.

**Figure 2:**
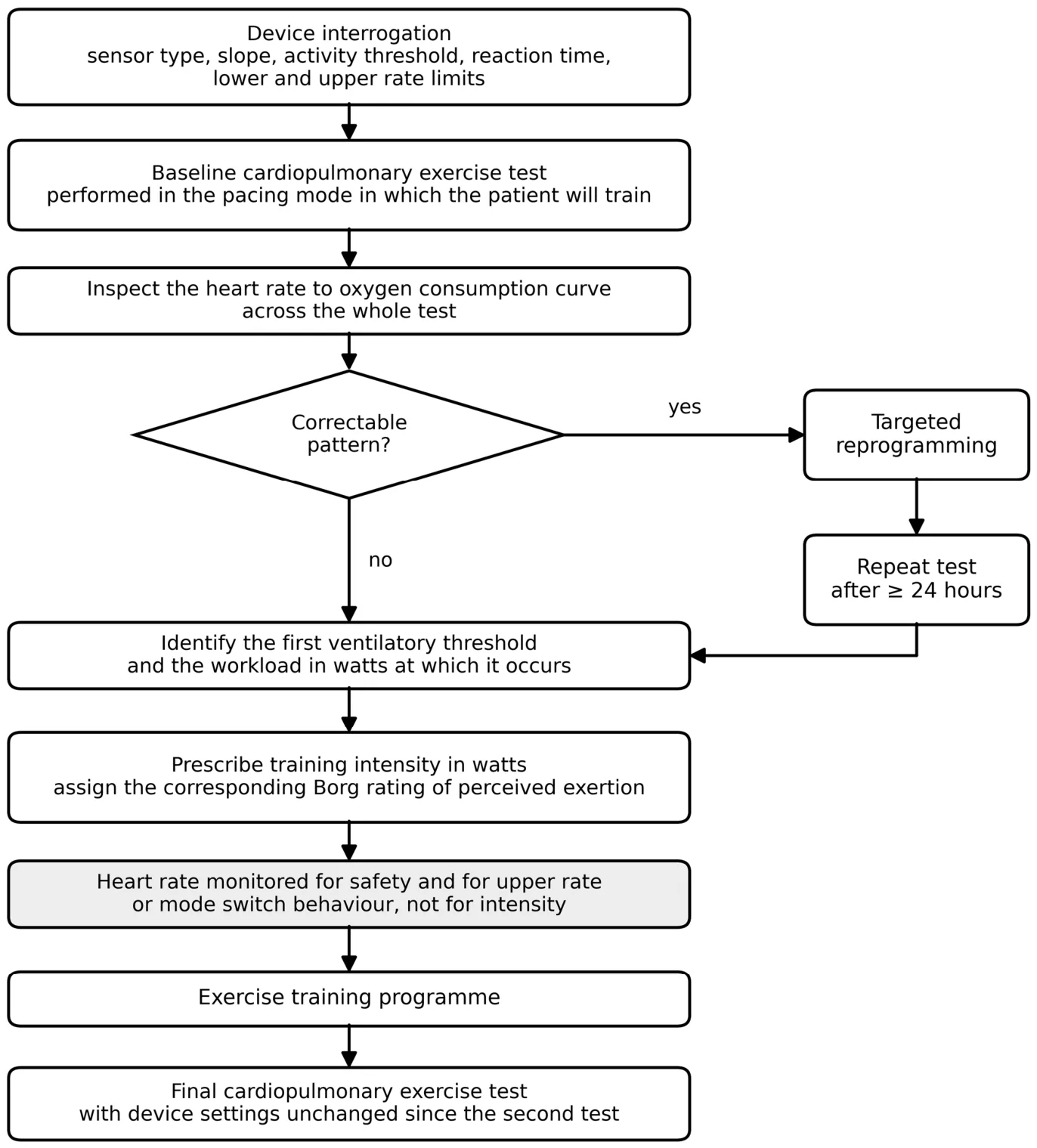
Proposed sequence linking cardiopulmonary exercise testing, device reprogramming and the prescription of exercise training in the pacemaker-wearing patient. The sequence is a framework derived from the reviewed mechanisms and has not been prospectively validated as a whole. The step shown on a grey background highlights the central practical point of the review: in the paced patient the heart rate is monitored for safety rather than used to set training intensity.

**Table 1:** Proposed minimum reporting set for a cardiopulmonary exercise test performed in a pacemaker-wearing patient.

Standard Cardiopulmonary Variables	Additions Specific to the Paced Patient
Peak oxygen consumption, absolute and per kilogram	Device model, pacing site and pacing mode during the test
Peak respiratory exchange ratio	Sensor type and programmed slope, threshold and reaction time
Oxygen consumption at the anaerobic threshold or a statement that it was not reached	Programmed lower and upper rate limits
VE/VCO_2_ slope	Percentage of paced and of sensed beats during the test
Oxygen uptake efficiency slope	Heart rate-to-oxygen consumption slope, expressed in bpm per mL/kg/min
Oxygen pulse at peak and its behaviour during the test	Heart rate at the anaerobic threshold
Maximum workload and exercise duration	Mode-switch episodes or upper rate behaviour observed during the test
Reason for stopping and peak Borg score	Beta-blocker treatment and whether it was maintained on the day of the test

**Table 2:** From the pattern observed in the cardiopulmonary exercise test to the corresponding programming action. The table represents a framework proposed by the authors and derived from the reviewed mechanisms; it is not a validated algorithm. The rows differ in the strength of their evidential support: adjustment of the sensor slope, the activity threshold and the upper rate limit rests on the physiological reasoning set out in the text, whereas the options concerning cardiac resynchronization and conduction-system pacing are complex clinical decisions governed by their own guidelines and by much stronger evidence, listed here only to indicate when they should be considered.

Pattern of the Heart Rate-to-Oxygen Consumption Relationship	Probable Mechanism	Programming Action	To Verify on the Repeat Test
Slope flat from the beginning of exercise	Rate response inactive or sensor gain set too low	Activate the rate-responsive function; increase the slope in stepwise fashion	Slope in bpm per mL/kg/min; heart rate at the anaerobic threshold
Rate remains at the lower value for the first minutes, then rises normally	Activity threshold set too high or reaction time too long	Lower the activity threshold; shorten the reaction time	Heart rate in the first three minutes of the incremental phase
Slope normal initially; abrupt flattening or fall in the final minutes	Upper rate limit reached, with Wenckebach behaviour or two-to-one block	Raise the upper rate limit; consider rate-adaptive shortening of the atrioventricular delay	Rate at which the fall occurred, compared with the programmed limit
Rate rises, then falls abruptly with a change in ventricular response	Mode switch triggered by an atrial arrhythmia during exercise	Review mode-switch thresholds and the discrimination algorithms; treat the arrhythmia	Device interrogation for mode-switch episodes during the test
Rate rises appropriately, but peak oxygen consumption does not improve or falls	Critical heart rate exceeded; stroke volume falling as rate rises	Reduce the maximum sensor-driven rate towards the critical heart rate	Oxygen pulse behaviour; peak oxygen consumption
High percentage of ventricular pacing with a wide paced QRS	Dyssynchrony from right ventricular pacing	Activate algorithms promoting intrinsic conduction; consider conduction-system pacing or resynchronization	Percentage of ventricular pacing; oxygen pulse; ejection fraction

**Table 3:** Proposed prescription sequence in the pacemaker-wearing patient.

Step	Action	Source of the Value
1	Interrogate the device and record the sensor type, slope, thresholds, reaction time, and lower and upper rate limits	Device interrogation before the test
2	Perform the baseline cardiopulmonary exercise test in the mode in which the patient will train	Section 4
3	Correct the programming if the heart rate-to-oxygen consumption curve shows a correctable pattern	Table 2
4	Consider repeating the test after reprogramming at an interval of at least 24 h—a pragmatic interval that has not been formally validated	Section 6.6
5	Identify the first ventilatory threshold and the workload in watts at which it occurs	V-slope method on the repeat test
6	Prescribe the continuous training intensity at that workload, expressed in watts	Threshold-based prescription
7	Assign a corresponding rating of perceived exertion for sessions performed without ergometry	Borg scale recorded during the test
8	Monitor heart rate during training for safety and for upper-rate or mode-switch behaviour; ventilatory thresholds and workload are preferred for setting of intensity	Section 7.3
9	Repeat the cardiopulmonary exercise test at the end of the programme, with the device settings unchanged between the two tests	Comparability of the two tests

## Data Availability

No new data were created or analysed in this study. Data sharing is not applicable to this article.

## Abbreviations

The following abbreviations are used in this manuscript:

CPET	Cardiopulmonary exercise testing
CRT	Cardiac resynchronization therapy
ICD	Implantable cardioverter-defibrillator
VO_2_	Oxygen consumption
VE/VCO_2_	Ventilatory equivalent for carbon dioxide
bpm	Beats per minute
MET	Metabolic equivalent of task
RPE	Rating of perceived exertion
